# Effect of lateral meniscectomy and osteochondral grafting of a lateral femoral condylar defect on contact mechanics: a cadaveric study in dogs

**DOI:** 10.1186/1746-6148-9-53

**Published:** 2013-03-22

**Authors:** Christina J Choate, Stanley E Kim, Caleb C Hudson, David Spreng, Antonio Pozzi

**Affiliations:** 1 Department of Small Animal Clinical Sciences, Comparative Orthopaedics and Biomechanics Laboratory, University of Florida, College of Veterinary Medicine, 2015 SW 16th Ave, Gainesville, FL 32610, USA; 2 Division of Small Animal Surgery and Orthopedics, Vetsuisse Faculty Bern, Department of Clinical Veterinary Medicine, University of Bern, Länggassstrasse 128, 3012, Bern, Switzerland

**Keywords:** Osteochondral autograft transfer, Contact mechanics, Pressure, Meniscus, Meniscectomy

## Abstract

**Background:**

Osteochondral autograft transfer (OAT) aims at restoring normal articular cartilage surface geometry and articular contact mechanics. To date, no studies have evaluated the contact mechanics of the canine stifle following OAT. Additionally, there are no studies that evaluated the role of the meniscus in contact mechanics following OAT in human or canine femorotibial joints. The objective of this study was to measure the changes in femorotibial contact areas (CA), mean contact pressure (MCP) and peak contact pressure (PCP) before and after osteochondral autograft transplantation (OAT) of a simulated lateral femoral condylar cartilage defect with an intact lateral meniscus and following lateral meniscectomy.

**Results:**

With an intact lateral meniscus, creation of an osteochondral defect caused a decrease in MCP and PCP by 11% and 30%, respectively, compared to the intact stifle (p < 0.01). With an intact meniscus, implanting an osteochondral graft restored MCP and PCP to 96% (p = 0.56) and 92% (p = 0.41) of the control values. Lateral meniscectomy with grafting decreased CA by 54% and increased PCP by 79% compared to the intact stifle (p < 0.01).

**Conclusions:**

OAT restored contact pressures in stifles with a simulated lateral condylar defect when the meniscus was intact. The lateral meniscus has a significant role in maintaining normal contact pressures in both stifles with a defect or following OAT. Meniscectomy should be avoided when a femoral condylar defect is present and when performing OAT.

## Background

Surgical strategies for the treatment of osteochondral defects of the stifle include debridement, curettage of the lesion and bone marrow stimulation with microfracture [1], or restorative procedures such as osteochondral autograft transfer (OAT) [2-4]. OAT consists of implanting an osteochondral graft into a recipient bed created at the site of the osteochondral defect [2-4]. In humans, the therapeutic use of osteochondral grafting has been demonstrated in multiple reports with successful short and long term outcomes reported, even in athletic patients [5-7]. In dogs, the use of OAT for treatment of osteochondrosis lesions of the lateral femoral condyle, medial aspect of the humeral condyle, and caudal humeral head has been performed with good functional outcomes reported [2-4].

Experimentally created osteochondral defects have been shown to alter femorotibial contact pressures in humans and dogs in several in vitro studies [8-10]. Similarly, naturally occurring defects such as osteochondrosis lesions may alter normal contact mechanics in affected joints. Alteration in cartilage contact mechanics may contribute to the pathogenesis of osteoarthritis and ultimately cause pain and poor function [10,11]. OAT aims at restoring normal articular cartilage surface geometry [3,5,10]. Several ex vivo studies have shown that by improving joint congruity, OAT can restore normal contact mechanics [11,12]. Koh et al. demonstrated that peak contact pressure was increased by creation of an osteochondral defect in swine stifles. Implantation of an osteochondral plug flush with the cartilage surface restored normal contact pressures, while implantation of an osteochondral plug in a recessed or elevated position both resulted in contact pressures higher than the control [11]. To date, no studies have evaluated the contact mechanics of the canine stifle following OAT. Additionally, there are no studies that evaluated the role of the meniscus in contact mechanics following OAT in human or canine femorotibial joints.

The purposes of this study were 1) to evaluate the effect of OAT on the contact area (CA), mean contact pressure (MCP) and peak contact pressure (PCP) of the lateral compartment of canine stifles with a simulated lateral femoral condylar osteochondral defect, 2) to evaluate the protective effect of the lateral meniscus in the presence of a lateral femoral osteochondral defect before and after OAT. We hypothesized that creation of a cartilage defect would significantly alter contact mechanics of the lateral compartment of the stifle and that OAT would restore the normal contact mechanics in the presence of an intact lateral meniscus. We hypothesized that removal of the lateral meniscus would significantly alter femorotibial contact mechanics, with or without the presence of a femoral condylar defect.

## Methods

### Specimen preparation and sensor placement

Eight unpaired pelvic limbs were harvested from large breed canine cadavers (body weight 28–35 kg) euthanatized for reasons unrelated to this study, as approved by the institution’s animal care and use committee (University of Florida IACUC # 201106858). Specimens were harvested within twelve hours of death. Radiographs of all limbs were obtained to ensure skeletal maturity and the absence of skeletal pathology. The skin, regional musculature, and stifle joint capsule were dissected and removed from the limbs. The portion of the femur proximal to the lesser trochanter, the distal tibial metaphysis, and the fibula 2 cm distal to the fibular head were ostectomized to facilitate placement in the testing apparatus. A 1.1 mm diameter Kirschner wire was drilled through the head of the fibula and proximal tibia, bent flush against the fibula and trimmed leaving 5–8 mm protruding from the fibula to stabilize the fibular head and insertion of the lateral collateral ligament. The stifles were wrapped in physiologic saline soaked towels and stored in a freezer at −20°C until they were thawed to room temperature for testing.

CA, PCP and MCP were recorded from a piezo-resistive pressure sensing system (Tekscan Inc., South Boston, USA). The sensor had two sensing areas of 30.9 × 12.0 mm and a thickness of 0.08 mm. Each sensing area contained 6 rows and 15 columns of sensing elements providing 90 sensels. The sensors had a pressure sensitivity of 0.01 MPa and a pressure range of 0.5 – 30.0 MPa. Each new sensor was conditioned then calibrated with a 10 mm-diameter indenter with an applied force of 15 N as described by the manufacturer’s guidelines. The contact map was recorded during the calibration and the calibration curve was calculated based on a software program provided by the sensor manufacturer immediately prior to testing of each specimen.

**Figure 1:**
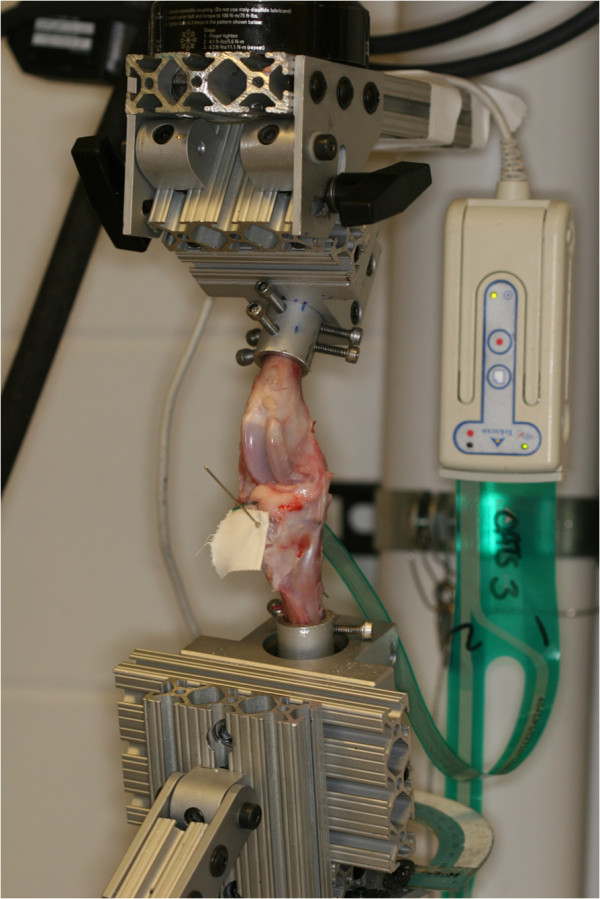
Illustration of the testing apparatus including stifle jig mounted in the material testing machine and digital pressure sensor positioned subjacent to lateral meniscus.

The potted specimens were thawed to room temperature while remaining moist, and wrapped in saline-soaked towels one hour prior to mechanical testing. Stifle inspection, creation of cartilage defects and lateral meniscectomies and sensor placement were performed via an approach to the stifle by osteotomy of the origin of the lateral collateral ligament. A 3.5 mm diameter bone tunnel was drilled through the femoral condyle, centered at the origin of the lateral collateral ligament of the stifle. A femoral osteotomy was performed to outline a block of bone that contained the entire origin of the lateral collateral ligament (Figure 1). The block of bone was freed from the underlying condyle using an osteotome allowing for the lateral collateral ligament to be reflected.

The lateral compartment was exposed by retracting the lateral collateral ligament, flexing and rotating the femoral condyles relative to the tibia. The sensor was gently manipulated into the space subjacent to the lateral meniscus. The sensor was advanced axially until its entire width was within the joint space. After positioning the sensor, the lateral collateral ligament osteotomy was fixed securely with a 3.5 mm screw, washer and nut. Sensor positioning was accomplished without causing any damage to the sensor by grasping it at its cranial and caudal edges.

### Mechanical testing

A testing fixture was used to mount the limb to the materials testing machine (858 Mini Bionix II, MTS Systems Corp., Eden Prairie, MN, USA). The end of the tibia was fixed to the platform with three degrees of freedom (cranial-caudal translation, medial-lateral translation and axial rotation). Once the stifle was positioned at 135 degrees of flexion, as measured using a goniometer, flexion-extension and rotation of the femur were constrained. For the tibia only flexion-extension was constrained, allowing tibial translation in the cranial-caudal and medial-lateral planes and axial rotation. Varus/valgus motion was allowed to account for changes in femorotibial congruity after meniscal excision during testing.

Mechanical testing was performed using an axial servohydraulic dynamic mechanical testing machine (858 MiniBionix II, MTS Systems Corp, Eden Prairie, MN). An axial force of 100 N was applied to each specimen over 5 seconds and then maintained for 11 seconds. Contact maps that allowed determination of instantaneous CA, MCP and PCP were recorded from each stifle in each of the 5 tested conditions. Contact maps were recorded 15 seconds into the loading protocol. The measurements were detected using a piezo-resistive pressure sensing system (Tekscan Inc., South Boston, USA).

### Surgical procedures

Each stifle was tested in five different conditions: intact condyle and intact lateral meniscus (control), lateral condylar defect with intact lateral meniscus (defect), OAT treated lateral condylar defect with intact lateral meniscus (OAT treated), OAT treated lateral condylar defect with lateral meniscectomy (OAT with meniscectomy), and lateral condylar defect with lateral meniscectomy (defect with meniscectomy). Surgical procedures were performed sequentially after testing each condition.

The 8-mm-diameter Osteochondral Autograft Transfer System (OATS) Donor Harvester Trephine (Arthrex Inc, Naples, FL USA) was used to obtain a 10-mm-deep osteochondral graft from the medial sulcus terminalis of the femur as previously described [2]. An 8-mm-diameter defect was created in the center of the weight-bearing articular surface of the femoral condyle. The stifle was hyperflexed and the Beath pin from the OATS kit was inserted to a depth of 20 mm at the apex of curvature of the lateral femoral condyle and perpendicular to the tangent of the femoral condyle contour. Using the Beath pin as a guide, the 8-mm-diameter OATS Cannulated Recipient Site Drill Bit was advanced to produce a 10-mm-deep defect in the articular cartilage and underlying subchondral bone, which would also serve as the recipient site for the autograft in the OAT treated conditions. The osteochondral graft obtained from the sulcus terminalis was trimmed as necessary to fit in the recipient site using a #10 scalpel blade, and set aside for use in the subsequent treatment conditions.

After recording a contact map for the defect condition, the autograft was inserted into the previously prepared recipient site and firmly seated using manual pressure. All grafts were measured and inserted such that the articular surface of the graft edges was flush with the articular cartilage of the recipient site. The femoral components were then re-secured to the jig and a contact map was recorded for the OAT treated condition. Without removing the femoral components from the jig, the intermeniscal ligament, lateral meniscotibial ligament, meniscofemoral ligament, and any attachments to the lateral collateral ligament and surrounding soft tissues were transected using a #11 scalpel blade. The lateral meniscus was removed, axial load was applied and a contact map was recorded for the OAT with meniscectomy condition. The femoral components were removed from the jig and the OAT autograft was removed from the recipient site. The femoral components were re-secured in the jig as previously described, axial load was applied and a contact map was recorded for the defect with meniscectomy condition. Consistent sensor positioning was confirmed in every specimen throughout testing by exposing both cranial and caudal poles of the meniscus before and after loading each condition.

### Data analysis

Statistical analysis was performed using a commercially available software system (SPSS). Contact area, MCP and PCP data were analyzed using multiple paired t-tests (2 tailed). Initially each treatment group was compared to the control group for a total of 4 comparisons. To evaluate the effect of lateral meniscectomy a fifth comparison was performed between the femoral condylar defect with intact lateral meniscus group and the femoral condylar defect with lateral meniscectomy group. A Bonferroni post hoc correction was utilized to correct our initially selected significant p value of 0.05 to account for multiple comparisons. The Bonferroni correction resulted in a p value of < 0.01 being set as significant.

## Results

### Effect of OAT

**Figure 2:**
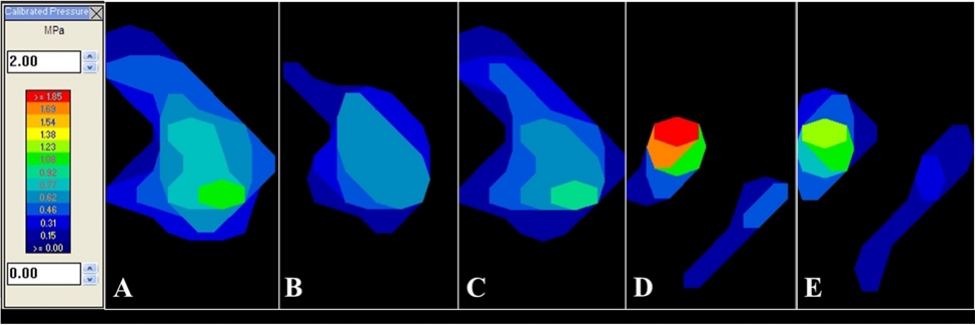
Pressure maps of the same stifle intact (A), with an 8 mm osteochondral defect and the meniscus intact (B), after OATS treatment of the defect with the meniscus intact (C), with an 8 mm osteochondral defect after meniscectomy (D), and after OATS treatment after meniscectomy (E).

Lateral CA, MCP, and PCP (mean ± SD) for each stifle condition are summarized in Table 1. Contact maps representative of each condition are provided in Figure 2. The creation of a condylar defect with an intact lateral meniscus significantly decreased MCP by 11% (p = 0.007) and PCP by 30% (p = 0.001) as compared to the control stifle. Treatment of the condylar defect with an OAT graft in the presence of an intact lateral meniscus restored the MCP and PCP to 96% (p = 0.558) and 92% (p = 0.412) of the control values, respectively. Contact area following OAT grafting was restored to 96% of the control values (0.176).

**Table 1:** Stifle contact mechanics – all groups

**Outcome measure**	**Contact area (mm**^**2**^**)**	**Peak contact pressure (MPa)**	**Mean contact pressure (MPa)**
Normal stifle (Control)	114.75 ± 17.51	1.67 ± 0.35	0.73 ± 0.12
Condylar defect, Meniscus intact	93.25 ± 20.50 (0.015)	1.20 ± 0.38* (0.001)	0.65 ± 0.15* (0.007)
OATS treated, meniscus intact	110.00 ± 19.33 (0.176)	1.80 ± 0.64 (0.412)	0.76 ± 0.20 (0.558)
Condylar defect, meniscus removed	53.25 ± 17.72* (0.000)	2.99 ± 1.33* (0.001)	1.01 ± 0.36 (0.026)
OATS treated, meniscus removed	51.63 ± 24.28* (0.002)	2.79 ± 1.50 (0.051)	1.16 ± 0.50 (0.039)

Values are mean ± standard deviation (p value) for contact area, mean contact pressure and peak contact pressure. Statistical comparisons are between each treatment group and the control group. Presence of an asterisk (*) indicates a significant difference between the treatment group and the control group (p ≤ 0.01).

### Effect of lateral meniscectomy

Removal of the lateral meniscus in the presence of a lateral femoral condylar defect resulted in a significant decrease in CA of 54% (p < 0.001) and an increase in PCP of 79% (p = 0.001) compared to the control stifle. Removal of the lateral meniscus with treatment of the lateral condylar defect by an OAT graft significantly decreased CA when compared to the control stifle (55% lower than control, [p = 0.002]), although PCP decreased to a value that was not significantly different from the control stifle (67% greater than control [p = 0.051]). Removal of the lateral meniscus in the presence of a lateral femoral condylar defect significantly decreased CA by 43% (p = 0.005) and increased MCP and PCP by 55% (p = 0.005) and 149% (p = 0.002) respectively as compared to the lateral femoral condylar defect with an intact lateral meniscus group (Table 2).

**Table 2:** Stifle contact mechanics – effect of meniscectomy

**Outcome measure**	**Contact area (mm**^**2**^**)**	**Peak contact pressure (MPa)**	**Mean contact pressure (MPa)**
Condylar Defect, Meniscus Intact	93.25 ± 20.50	1.20 ± 0.38	0.65 ± 0.15
Condylar Defect, Meniscus Removed	53.25 ± 17.72* (0.005)	2.99 ± 1.33* (0.002)	1.01 ± 0.36* (0.005)

Values are mean ± standard deviation (p value) for contact area, mean contact pressure and peak contact pressure. Statistical comparisons are between the two treatment groups. Presence of an asterisk (*) indicates a significant difference between the two treatment groups (p ≤ 0.01).

## Discussion

In this biomechanical study, the first goal was to determine the effect of a lateral femoral condylar defect treated with OAT on stifle contact mechanics. The second goal of the study was to evaluate the contribution of the lateral meniscus to stifle contact mechanics in the presence of a lateral condylar defect. Based on our results, OAT restored normal femorotibial contact mechanics in the lateral compartment of a stifle with a lateral condylar defect. Additionally, the lateral meniscus was found to have a significant role in distributing pressure across the lateral compartment in the presence of an osteochondral defect. The significant increase in PCP measured after lateral meniscectomy demonstrates that the intact lateral meniscus has a protective effect on femorotibial contact pressure in the presence of a condylar defect. Although this cadaveric study cannot simulate in vivo conditions, these findings support the use of OAT in dogs with osteochondrosis lesions of the lateral femoral condyle. Furthermore, the significant contribution of the lateral meniscus to contact mechanics in joints with a lateral condylar defect and after OAT treatment suggest that a lateral meniscectomy should be avoided in dogs with osteochondrosis lesions of the lateral femoral condyle.

In this canine model an osteochondral defect in the weight bearing portion of the lateral femoral condyle decreased rather than increased stifle peak contact pressures, although the decrease was not statistically significant. This result differs from previous studies in canine and human knees, which reported up to a 192% increase in peak contact pressures when a condylar defect was created [8,11,13,14]. In the normal lateral compartment, the region of peak pressure may have been located at the most distal portion of the femoral condyle that contacted either the thin axial margin of the lateral meniscus or the tibial plateau directly; removal of this region of condyle by creating our defect may have shifted the peak pressure to the rim of the defect, where load was transmitted more uniformly through a more peripheral, thicker portion of the meniscus. Our analysis did not include defining the precise location of the peak pressure to confirm this potential explanation. Another reason for the lack of higher pressures with the defect may be the low axial loads used in this study. Greater axial loads might have resulted in higher pressures by increasing contact at the rim of the condylar defect. The rim stress effect around an osteochondral lesion can be influenced by defect size, the size of the defect relative to the affected femoral condyle, and individual variations in femoral condyle and tibial plateau geometry [8,13]. Our findings are similar to a cadaveric study that evaluated the effect of OAT on talar defects in human ankles and reported contact maps without a discernable rim stress effect [12]. Finally, the lack of a discernable rim stress effect in our study may have been the result of the relative low resolution of the digital pressure sensor, which may have not be sufficient for characterizing this small size defect [12,13].

OAT treatment restored normal stifle contact mechanics in the presence of an intact lateral meniscus. The results of OAT treatment in our study are similar to the results of several biomechanical studies in the human literature, which demonstrated that the implantation of an osteochondral autograft restored normal knee contact pressures [11-14]. It has been shown that untreated experimentally induced osteochondral lesions of 6 mm diameter in canine femoral condyles heal with fibrocartilage that do not maintain the same biomechanical properties as articular cartilage, and do not restore normal contact mechanics [9]. It has been suggested that high-pressure gradients through the articular cartilage surrounding a defect could significantly impair chondrocyte production, matrix production, and maturation of granulation tissue at the defect site [8]. Restoration of the normal joint contact pressures and contact area with and around an osteochondral autograft via the OAT procedure may eliminate these abnormal pressure gradients and promote healing at the graft site.

There are no previous human or animal biomechanical studies evaluating the contribution of the meniscus to contact pressure of a condylar defect before and after treatment with OAT. We designed the study to perform all treatments sequentially in each tested joint, so that we could use the same control for all treatments. This design carried the limitation of not including a control for the analysis of meniscal treatments (meniscectomy - no osteochondral defect), which did not allow us to run a two-way ANOVA repeated measure, necessary to analyze two factors, meniscus status and defect. Instead we selected multiple t-tests with a Bonferroni correction to allow multiple comparisons while reducing false positives.

In this study the removal of the lateral meniscus caused a 149% and 55% increase in PCP in the stifle with an untreated lateral condylar defect and with an OAT treated lateral condylar defect, respectively. Previous studies have reported 88% increases in PCP following medial meniscectomy [15,16]. These changes are consistent with human studies that have previously reported increases in medial and lateral compartment contact stress of 100% and 200 – 300%, respectively after medial or lateral meniscectomy [17]. The meniscus responds to load mainly by compression. After meniscectomy has been performed, the lack of a spacer between the femur and tibia causes focal contact between the curved femoral and tibial condyles. In our study the lateral meniscus likely acted as a spacer between the rim of the defect and the tibial surface since the lateral meniscus covered the majority of our simulated osteochondral defect. The clinical relevance of this finding may be multifold. Abaxial osteochondrosis defects entirely in contact with the lateral meniscus at all flexion angles may be amenable to conservative treatment. More axial defects, which are not completely covered by meniscus, may be better candidates for OAT. Additionally, based on our results a conservative strategy is recommended when managing lateral meniscal tears in stifles with osteochondrosis lesions of the lateral femoral condyle [13,18-20].

There are several limitations to this study. Testing was performed by axially loading stifles at a static flexion angle and cannot fully replicate the complex shear and rotational forces to which the condyle is exposed in vivo, nor does it take into account the biological response of the local joint environment. Our study design did not allow us to incorporate a group which received a lateral meniscectomy in the presence of an intact femoral condyle, this group would have been a more appropriate control to compare with the meniscectomy defect group than our normal stifle control group.

## Conclusions

In conclusion, OAT of a lateral femoral osteochondral defect restored normal contact mechanics with an intact lateral meniscus but failed to restore normal contact mechanics post-meniscectomy. In addition, the lateral meniscus had an important role in contact pressure regulation when the condylar defect was untreated. Based on our results we recommend OAT for the treatment of lateral condylar defects of the stifle, and a conservative approach to lateral meniscal resection when an osteochondral defect is present.

## Competing interests

None of the authors believe their interpretation or presentation of the data was influenced by any financial competing interests. Dr Antonio Pozzi is a paid consultant with Arthrex, received honoraria in connection with sponsored Continuing Education seminars with Arthrex and received funding from Arthrex for previous projects. This study was funded with faculty start-up money.

## Authors’ contributions

CJC and AP conceived and designed the study; CJC and CCH collected the data; SEK and CCH performed the statistical analysis; AP, SEK, CCH and DS interpreted the results; CJC and AP drafted the manuscript; all authors read, contributed to and approved the final manuscript.
